# Motor- and cognition-related safety of pimavanserin in patients with Parkinson's disease psychosis

**DOI:** 10.3389/fneur.2022.919778

**Published:** 2022-10-05

**Authors:** Victor Abler, Cecilia Brain, Clive Ballard, Ana Berrio, Bruce Coate, Alberto J. Espay

**Affiliations:** ^1^Acadia Pharmaceuticals Inc, San Diego, CA, United States; ^2^University of Exeter Medical School, Exeter, United Kingdom; ^3^Gardner Family Center for Parkinson's Disease and Movement Disorders, Department of Neurology, University of Cincinnati, Cincinnati, OH, United States

**Keywords:** Parkinson's disease, pimavanserin, cognition, motor function, psychosis

## Abstract

**Background:**

Pimavanserin, a selective 5-HT_2A_ inverse agonist/antagonist, is the only treatment approved by the US Food and Drug Administration for hallucinations and delusions associated with Parkinson's disease (PD) psychosis.

**Aim:**

We aimed to evaluate motor- and cognition-related safety in pimavanserin-treated patients with PD psychosis.

**Methods:**

This analysis included patients with PD psychosis treated with pimavanserin 34 mg from a pooled analysis of 3 randomized, double-blind, placebo-controlled, 6-week studies [NCT00477672 (study ACP-103-012), NCT00658567 (study ACP-103-014), and NCT01174004 (study ACP-103-020)] and a subgroup of patients with PD dementia with psychosis from HARMONY (NCT03325556), a randomized discontinuation study that included a 12-week open-label period followed by a randomized double-blind period of up to 26 weeks. Motor- and cognition-related safety were examined.

**Results:**

The pooled analysis included 433 randomized patients (pimavanserin, 202; placebo, 231). Least squares mean (standard error [SE]) change from baseline to week 6 Unified Parkinson's Disease Rating Scale (UPDRS) II + III score was similar for pimavanserin [−2.4 (0.69)] and placebo [−2.3 (0.60)] (95% Confidence Interval [CI]:−1.9, 1.6). The change from baseline to week 6 for UPDRS II and UPDRS III scores was similar between groups. In the HARMONY open-label period, 49 patients with PD dementia with psychosis were treated with pimavanserin 34 mg, 36 of whom were randomized in the double-blind period (pimavanserin, 16; placebo, 20). In the open-label period, the mean (SE) change from baseline to week 12 (*n* = 39) Extra-Pyramidal Symptom Rating Scale (ESRS-A) score was −1.7 (0.74); in the double-blind period, the results were generally comparable between the pimavanserin and placebo arms. The change from baseline in Mini-Mental State Examination (MMSE) score was also comparable between pimavanserin- and placebo-treated patients in HARMONY [open-label (*n* = 37): mean (SE) change from baseline to week 12, 0.3 (0.66)]. Rates of motor- and cognition-related adverse events were similar between pimavanserin and placebo in both analyses.

**Conclusions:**

Pimavanserin 34 mg was well tolerated and did not yield a negative impact on motor- or cognition-related function in patients with PD psychosis.

## Introduction

Hallucinations and delusions associated with Parkinson's disease (PD) exacerbate the clinical burden of PD for patients and caregivers (1–3). Patients with PD psychosis have a decreased quality of life and increased morbidity and healthcare resource utilization, relative to those with PD without psychosis (2, 4, 5). Caregivers of patients with PD psychosis also report a higher burden than caregivers of patients with PD without psychosis (3, 6). Importantly, psychosis increased the risk of progression to nursing home placement and death in PD patients (7–10).

Off-label antipsychotics are commonly prescribed to treat PD psychosis. Adverse events (AEs) related to antipsychotics can considerably impair the overall well-being and functioning of patients with PD psychosis (11–14). Particularly important for patients with PD, most off-label antipsychotics have antagonist activity at the dopamine D_2_ receptor and can exacerbate the motor symptoms associated with PD (14, 15). Parkinsonism (extrapyramidal symptoms) and worsening motor function are commonly reported AEs with antipsychotic use that may be particularly important in this context (11, 13, 16, 17). Motor impairment associated with off-label use of antipsychotics in older adults has a negative impact on activities of daily living, such as bathing, dressing, and walking, and on instrumental activities of daily living, such as managing medication, house cleaning, and shopping (18). In addition to motor impairments, off-label antipsychotics have also been shown to exacerbate cognitive function decline in patients with dementias (13, 19). A meta-analysis of clinical trials of atypical antipsychotics in patients with Alzheimer's disease and other dementias demonstrated that olanzapine, risperidone, and aripiprazole were associated with a significant worsening in Mini-Mental State Examination (MMSE) scores relative to placebo (19).

Pimavanserin, a selective serotonin (5-HT_2A_) antagonist/inverse agonist (20), is the only treatment approved by the US Food and Drug Administration for hallucinations and delusions associated with PD psychosis. This approval was based on data from clinical trials indicating improvement across select PDP measures (20, 21). The objective of this analysis was to describe safety data for motor- and cognition-related function and AEs of interest available from placebo-controlled studies of pimavanserin 34 mg in patients with PD psychosis to inform healthcare providers considering the risk–benefit profile of antipsychotic treatment of frail and elderly patients with PD psychosis.

## Materials and methods

### Study design and patients

Safety findings were analyzed in a pooled sample of 3 randomized, double-blind, placebo-controlled studies of pimavanserin in patients with PD psychosis over 6 weeks [NCT00477672 (study ACP-103-012, or 012), NCT00658567 (study ACP-103-014, or 014), and NCT01174004 (study ACP-103-020, or 020)] and in a subgroup of patients with PD dementia with psychosis in HARMONY (NCT03325556), a randomized discontinuation trial in patients with dementia-related psychosis. The study designs and primary results of study 020 and HARMONY have been previously published (22, 23).

#### Motor function evaluation

Motor function was evaluated using the Unified Parkinson's Disease Rating Scale parts II and III (UPDRS II + III) in studies 012, 014, and 020 (24). UPDRS II measures the patient's experience of motor function in daily life, such as speech, eating, walking, engaging in activities, and experiences of tremor or freezing. UPDRS III is an independent evaluation of motor signs of PD in the patient, such as posture, tremor, bradykinesia, gait, and speech. Higher scores on the UPDRS indicate worsened function, and lower scores indicate improved function. UPDRS II + III were administered at baseline and at week 6 in all 3 studies; change from baseline to week 6 was analyzed in the pooled sample.

The Extrapyramidal Symptom Rating Scale-Abbreviated (ESRS-A) was used to evaluate motor function in HARMONY. The ESRS-A is an assessment developed to measure drug-induced movement disorders by evaluating Parkinsonism, dyskinetic movements, and signs of tardive dyskinesia (25). Similar to the UPDRS, higher scores indicate worsened motor function. ESRS-A was assessed at baseline, week 12 of the open-label period, and each study visit during the double-blind period (i.e., weeks 13, 14, 16, 18, 22, 26, 30, 34, and 38).

Motor-related treatment-emergent adverse events (TEAEs) of interest were examined by preferred terms across studies. Standardized Medical Dictionary for Regulatory Activities queries for Extrapyramidal syndrome, which consisted of subqueries for akathisia, dyskinesia, dystonia, and Parkinson-like events (71 preferred terms) and sedation (6 preferred terms), were used to identify preferred terms of interest (Supplementary Table 1). Falls and orthostatic hypotension were also examined.

#### Cognitive function evaluation

Cognitive function was a prespecified safety assessment in HARMONY. Cognitive abilities were evaluated using the MMSE, which was administered at baseline and at each study visit during the open-label and double-blind periods of HARMONY. For the studies (012, 014, and 020) included in the pooled analysis, MMSE was not collected after the screening assessment (i.e., during pimavanserin treatment). Lower scores on the MMSE indicate cognitive decline.

Cognition-related TEAEs were assessed in the pooled sample and in HARMONY. Cognition-related TEAEs were examined across studies using a Standardized Medical Dictionary for Regulatory Activities query for “Cognitive and attention disorders and disturbances.” Confusional state was also examined for a total of 14 preferred terms (Supplementary Table 2).

#### Description of individual studies and patient populations

Studies 012, 014, and 020 were phase 2b/3 or 3, randomized, double-blind, placebo-controlled trials of pimavanserin in patients with hallucinations and/or delusions associated with PD psychosis (23). The pooled analysis included patients from all 3 studies treated with pimavanserin 34 mg once daily or placebo. While some patients in these trials were administered different dosages of pimavanserin, this analysis included those who took 34 mg, as this dose is approved as a part of the drug label for PDP (26). Since no patients were treated with pimavanserin 34 mg in 014, only patients receiving placebo from this study were included in the analysis.

The studies enrolled patients aged ≥40 years with PD and visual or auditory hallucinations and/or delusions. Enrolled patients were required to have a clinical diagnosis of PD with a duration of at least 1 year, and symptoms of psychosis were to have developed after the PD diagnosis and have been present during the 4 weeks before study screening. All patients were on a stable dose of anti-Parkinson medication for at least 1 month before the first study visit. Dose remained fixed for the duration of the trial. Patients with an MMSE score ≥21 at screening were eligible.

In study 012, patients were randomized to receive pimavanserin 34 mg (*n* = 98), pimavanserin 8.5 mg (*n* = 99), or placebo (*n* = 98) for 6 weeks. In study 014, patients were randomized to receive pimavanserin 8.5 mg (*n* = 41), pimavanserin 17 mg (*n* = 41), or placebo (*n* = 39) for 6 weeks. In study 020, patients were randomized to receive pimavanserin 34 mg (*n* = 104) or placebo (*n* = 94) for 6 weeks (23). This analysis included patients treated with pimavanserin 34 mg or placebo.

HARMONY was a phase 3 randomized discontinuation trial of pimavanserin in patients with dementia-related psychosis (22). The HARMONY study included patients with psychosis related to Alzheimer's disease, Parkinson's disease dementia, dementia with Lewy bodies, frontotemporal dementia, or vascular dementia. This analysis included a subgroup of only patients with PD psychosis, and is focused on safety events in PDP, rather than the larger heterogeneous population included in HARMONY. Patients receiving pimavanserin 34 mg had a clinical diagnosis of possible or probable PD dementia (*n* = 49), Alzheimer's dementia (*n* = 243), dementia with Lewy bodies (*n* = 27), frontotemporal-spectrum disorders (*n* = 7), and/or vascular dementia (*n* = 36) and psychotic symptoms (hallucinations and/or delusion) for at least 2 months before screening. Patients aged ≥50 and ≤ 90 years with MMSE scores of ≥6 and ≤ 24 at screening were eligible.

HARMONY included a 12-week open-label period followed by a randomized double-blind period of up to 26 weeks. All enrolled patients initiated pimavanserin 34 mg in the open-label period. A dose reduction to 20 mg daily based on tolerability was permitted from weeks 1 to 4, after which the dose remained fixed for the remainder of the open-label period. Patients with a sustained response to pimavanserin were randomized 1:1 to continue pimavanserin or receive placebo in the double-blind period. This subgroup analysis included patients with PD dementia with psychosis symptoms in HARMONY who were treated with pimavanserin 34 mg (*n* = 65) or placebo (*n* = 20). (Patients whose pimavanserin dose was reduced to 20 mg were excluded from the analysis).

### Statistical analysis

Change from baseline in UPDRS II + III score for the pooled analysis was calculated as least squares mean (LSM) from a mixed-effect model repeated measure model. Change from baseline in ESRS-A score and MMSE score in HARMONY was analyzed using descriptive statistics. Motor- and cognition-related TEAEs were analyzed using descriptive statistics.

## Results

### Patients

The pooled analysis included 202 patients treated with pimavanserin 34 mg and 231 patients treated with placebo (Table 1). Mean age [standard deviation (SD)] was comparable between pimavanserin-treated patients and placebo-treated patients [71.1 (7.33) vs. 71.5 (8.84) years]. There were statistically more male patients in the pimavanserin 34-mg group than in the placebo group (71.3 vs. 58.0%; *p* = 0.005). Most patients in both groups were White. At baseline, mean (SD) UPDRS Parts II + III and MMSE scores were similar between groups.

**Table 1:** Baseline characteristics of patients in studies 012, 014, and 020 (pooled) and of patients with PD dementia with psychosis in HARMONY.

	**Studies 012, 014, and 020**		**HARMONY**		
	**Pimavanserin 34 mg (*n =* 202)**	**Placebo (*n =* 231)**	**Open-label pimavanserin 34 mg (*n =* 49)**	**Double-blind pimavanserin 34 mg (*n =* 16)**	**Double-blind placebo (*n =* 20)**
Age (y), mean (SD)	71.1 (7.33)	71.5 (8.84)	72.6 (7.59)	69.6 (7.12)	72.3 (8.61)
**Sex**, ***n*** **(%)**					
Men	144 (71.3)	134 (58.0)	30 (61.2)	10 (62.5)	12 (60.0)
Women	58 (28.7)	97 (42)	14 (38.9)	6 (37.5)	8 (40.0)
**Race**, ***n*** **(%)**					
White	183 (90.6)	209 (90.5)	47 (100)	16 (100)	19 (100)
Black	2 (1.0)	3 (1.3)	0 (0.00)	0 (0.00)	0 (0.00)
Asian	11 (5.4)	12 (5.2)	0 (0.00)	0 (0.00)	0 (0.00)
Other	6 (3.0)	7 (3.0)	0 (0.00)	0 (0.00)	0 (0.00)
**Ethnicity**, ***n*** **(%)**					
Hispanic	6 (3.0)	5 (2.2)	6 (12.8)	3 (18.8)	1 (5.3)
Non-Hispanic	196 (97.0)	226 (97.8)	41 (87.2)	15 (81.3)	18 (94.7)
UPDRS II + III, mean (SD)	52.0 (19.26)	52.5 (19.32)	N/A	N/A	N/A
UPDRS II, mean (SD)	18.3 (6.85)	18.5 (7.17)	N/A	N/A	N/A
UPDRS III, mean (SD)	33.6 (14.40)	34.0 (13.99)	N/A	N/A	N/A
ESRS-A, mean (SD)			26.2 (13.24)	27.4 (15.96)	26.3 (14.03)
MMSE, mean (SD)	26.0 (2.66)	26.4 (2.54)	18.9 (5.18)	19.6 (5.03)	19.3 (5.79)

Number of patients with non-missing values used as the denominator for each group.

ESRS-A, Extrapyramidal Symptom Rating Scale-Abbreviated; MMSE, Mini-Mental State Examination; N/A, not applicable; PD, Parkinson's disease; SD, standard deviation; UPDRS, Unified Parkinson's Disease Rating Scale.

HARMONY enrolled 59 patients with PD dementia with psychosis, 49 of whom were stabilized on pimavanserin 34 mg in the open-label period (Table 1); 36 patients on pimavanserin 34 mg throughout the open-label period were randomized to pimavanserin 34 mg (*n* = 16) or placebo (*n* = 20). At open-label baseline, patients were a mean (SD) 72.6 (7.59) years of age and 61.2% (*n* = 30) were male; 100% (*n* = 49) of patients were White. Mean (SE) ESRS-A was 26.2 (1.89), and mean (SE) MMSE was 18.9 (0.74) at open-label baseline; mean ESRS-A and MMSE scores were balanced between pimavanserin and placebo groups at double-blind baseline.

### Motor function evaluation

#### Pooled analysis of studies 012, 014, and 020

**Figure 1:**
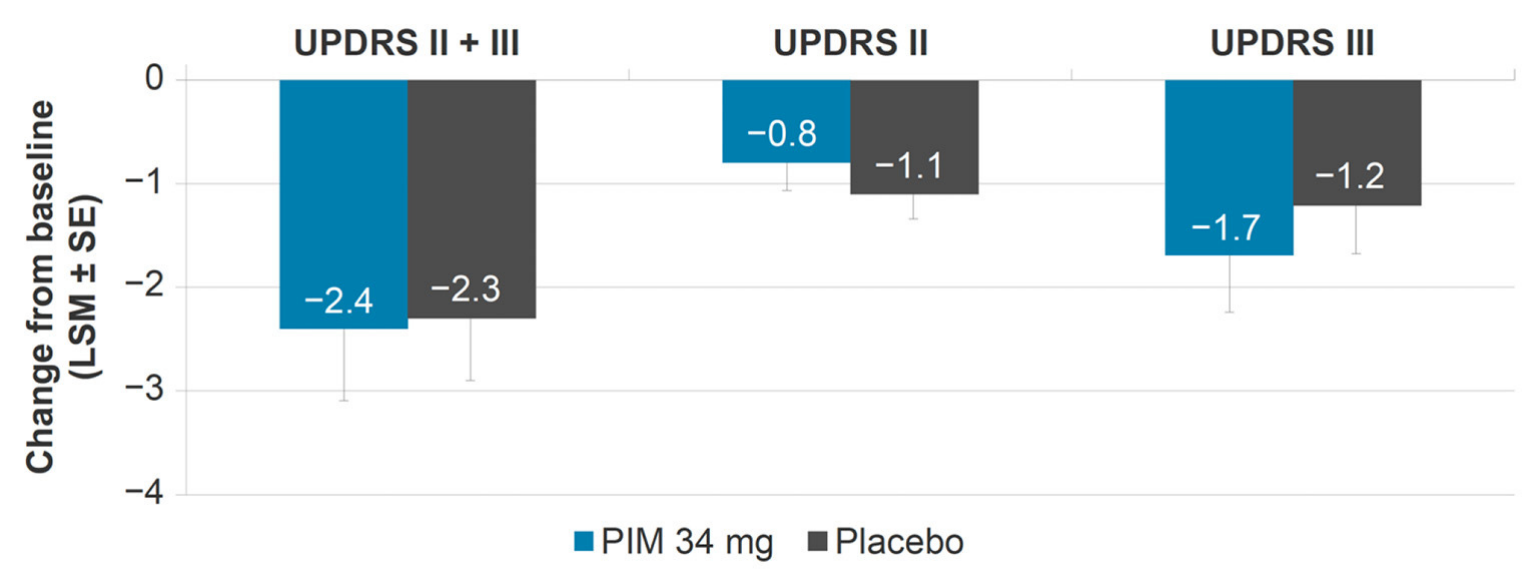
Change from baseline in UPDRS II, III, and II + III, pooled studies 012, 014, and 020 (pimavanserin 34 mg). LSM, least squares mean; PIM, pimavanserin; SE, standard error; UPDRS, Unified Parkinson's Disease Rating Scale.

The UPDRS II + III LSM [standard error (SE)] change from baseline to week 6 was similar between pimavanserin 34 mg [−2.4 (0.69)] and placebo [−2.3 (0.6)] [95% Confidence Interval (CI): −1.9, 1.6] (Figure 1). Activities of daily living (UPDRS II) and motor examination (UPDRS III) scores were also similar between groups when evaluated independently. The UPDRS II LSM (SE) change from baseline to week 6 was −0.8 (0.28) in pimavanserin-treated patients and −1.1 (0.24) in placebo-treated patients (95% CI: −0.4, 1.0). The UPDRS III LSM (SE) change from baseline to week 6 was −1.7 (0.54) in pimavanserin-treated patients and −1.2 (0.47) in placebo-treated patients (95% CI: −1.8, 0.9).

Falls occurred in 6.4% (*n* = 13) of pimavanserin-treated patients and 9.1% (*n* = 21) of placebo-treated patients (Table 2). Incidence of orthostatic hypotension TEAEs was numerically lower in pimavanserin-treated patients [1.0% (*n* = 2)] than placebo-treated patients [5.2% (*n* = 12)]. The rate of orthostatic hypotension assessed by vital sign criteria was also numerically lower in pimavanserin- than placebo-treated patients [pimavanserin, 29.6% (*n* = 58); placebo, 38.4% (*n* = 88)]. Motor-related events (Parkinson-like events) occurred at similar rates in both groups [pimavanserin 4.5% (*n* = 9); placebo 6.1% (*n* = 14)]; however, gait disturbance occurred at a higher rate in the pimavanserin group [2.5% (*n* = 5)] vs. placebo [0.4% (*n* = 1)].

**Table 2:** Motor-related TEAEs, pooled studies 012, 014, 020 (pimavanserin 34 mg).

**Events, *n* (%)**	**Pooled studies 012, 014, and 020**			
	**Pimavanserin (*n =* 202)**	**Placebo (*n =* 231)**
Fall	13 (6.4)	21 (9.1)
**Orthostatic hypotension**		
Vital sign criteria^a^	58/196 (29.6)	88/229 (38.4)
TEAE PT orthostatic hypotension	2/202 (1.0)^b^	12/231 (5.2)
Either vital sign criteria^a^ or TEAE PT orthostatic hypotension	58/202 (28.7)^b^	95/231 (41.1)
Parkinson-like events	9 (4.5)	14 (6.1)
Gait disturbance	5 (2.5)	1 (0.4)
Parkinson's disease	3 (1.5)	1 (0.4)
Tremor	1 (0.5)	4 (1.7)
Freezing phenomenon	1 (0.5)	2 (0.9)
Hypertonia	1 (0.5)	0 (0.0)
Muscle rigidity	1 (0.5)	0 (0.0)
Musculoskeletal stiffness	1 (0.5)	1 (0.4)
Parkinsonism	0 (0.0)	0 (0.0)
Bradykinesia	0 (0.0)	1 (0.4)
Drooling	0 (0.0)	1 (0.4)
Parkinsonian gait	0 (0.0)	1 (0.4)
Sedation-related events	13 (6.4)	12 (5.2)
Sedation	0 (0.0)	0 (0.0)
Somnolence	5 (2.5)	6 (2.6)
Fatigue	5 (2.5)	5 (2.2)
Asthenia	3 (1.5)	1 (0.4)
Lethargy	2 (1.0)	0 (0.0)
Hypersomnia	0 (0.0)	0 (0.0)

a Orthostatic hypotension was defined as a decrease of ≥20 mmHg in systolic blood pressure (SBP) OR a decrease of ≥15 mmHg in diastolic blood pressure (DBP), OR an increase of ≥20 bpm in pulse rate (PR); each measured from 5 min supine to 1 min standing at the same visit.

b Met p < 0.05 level of significance using Fisher's Exact test by comparing the incidence rate for each pimavanserin group vs. placebo.

PT, preferred term; TEAE, treatment-emergent adverse events.

#### HARMONY

**Figure 2:**
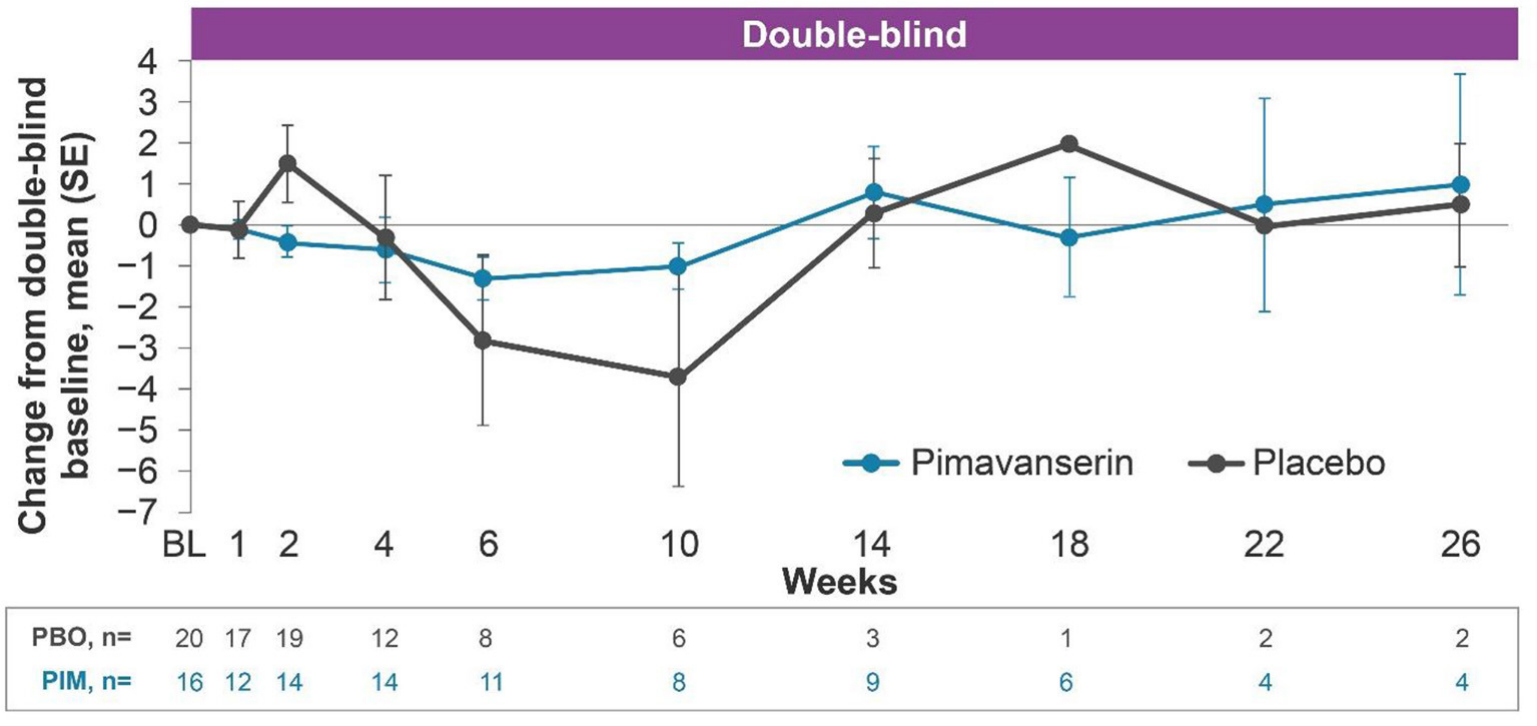
Change from baseline ESRS-A total score, HARMONY PD dementia with psychosis (pimavanserin 34 mg) subgroup. PD dementia with psychosis pimavanserin 34-mg subgroup as of study end date data cutoff of Oct, 30 2019. BL, baseline; ESRS-A, Extrapyramidal Symptom Rating Scale-Abbreviated; PBO, placebo; PD, Parkinson's disease; PIM, pimavanserin; SE, standard error.

In the open-label period of HARMONY, mean (SE) change from baseline to week 12 (*n* = 39) ESRS-A score was −1.7 (0.74). In the double-blind period, the results were generally comparable between the pimavanserin and placebo arms (Figure 2). In the open-label period of HARMONY (*N* = 49), 1 patient (2.0%) reported dysphonia, 1 patient (2.0%) reported psychomotor hyperactivity, and 3 patients (6.1%) reported falls. Two patients (4.1%) reported orthostatic hypotension. Somnolence [*n* = 4 (8.2%)], fatigue [*n* = 2 (4.1%)], and asthenia [*n* = 1 (2.0%)] were the only sedation-like events reported. In the double-blind period of HARMONY (pimavanserin, *n* = 16; placebo, *n* = 20), dyskinesia, tremor, orthostatic hypotension, hypersomnia, and somnolence were reported by 1 (5.0%) placebo-treated patient each. No motor-related AEs were reported in pimavanserin-treated patients in the double-blind period.

### Cognition-related function evaluation

#### Pooled analysis of studies 012, 014, and 020

Confusional state occurred in 5.9% (*n* = 12) of pimavanserin-treated patients and 2.6% (*n* = 6) of placebo-treated patients. Cognitive disorder was reported by 1 (0.4%) placebo-treated patient.

#### HARMONY

**Figure 3:**
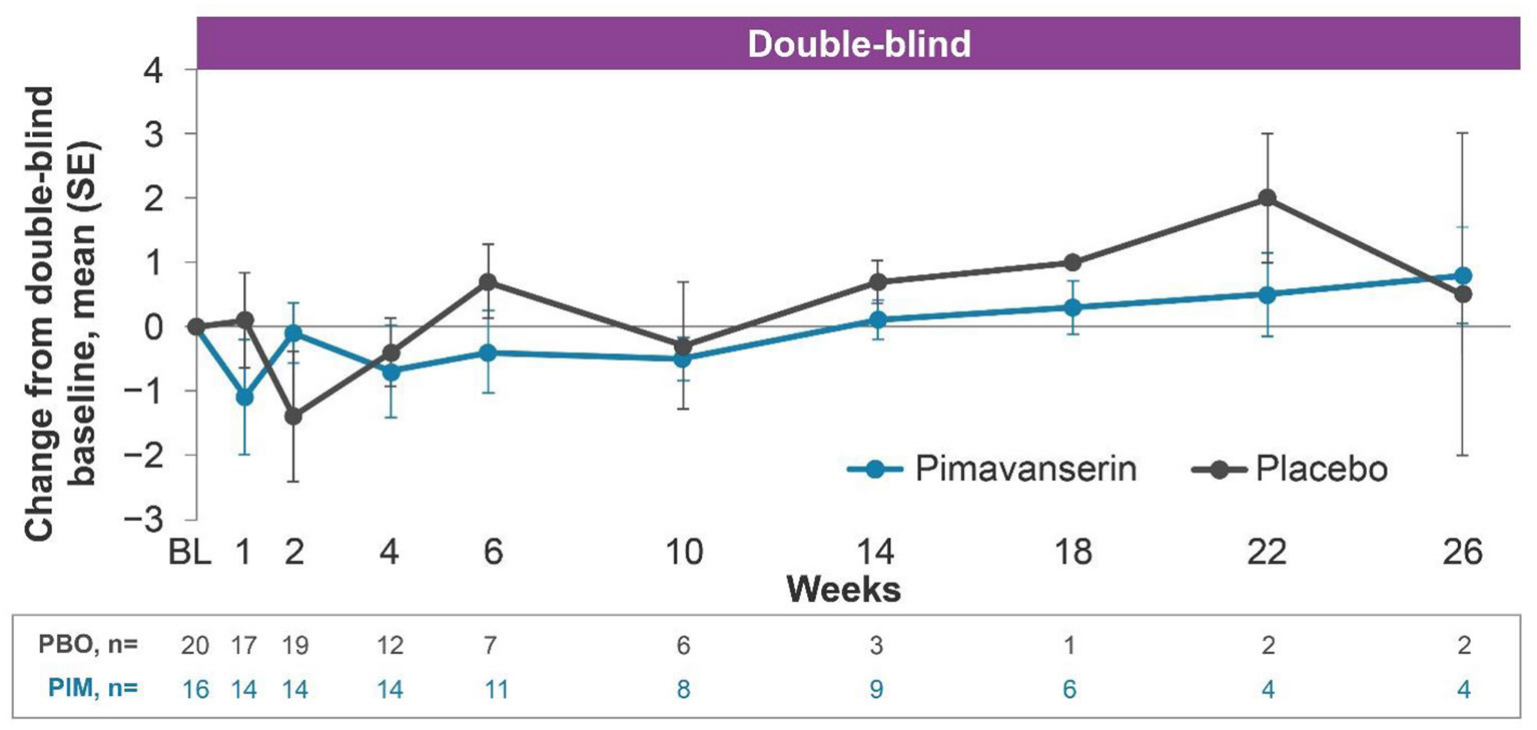
Change from baseline MMSE score, HARMONY PD dementia with psychosis (pimavanserin 34 mg) subgroup. PD dementia with psychosis pimavanserin 34-mg subgroup as of study end date data cutoff of Oct, 30 2019. BL, baseline; MMSE, Mini-Mental State Examination; PBO, placebo; PD, Parkinson's disease; PIM, pimavanserin; SE, standard error.

In the open-label period, mean (SE) change from baseline to week 12 MMSE score was 0.3 (0.66) (Figure 3). In the double-blind period, the results were generally comparable between the pimavanserin and placebo arms. In the open-label period of HARMONY, confusional state was reported by 4.1% (*n* = 2) of patients. No cognitive-related TEAEs were reported during the double-blind period of HARMONY.

## Discussion

Pimavanserin was well tolerated in patients with PD psychosis, including those with PD dementia with psychosis from the HARMONY study. There was no negative impact of pimavanserin 34 mg on the UPDRS II + III, ESRS-A, or MMSE, suggesting no worsening of motor- and cognition-related-function or cognition. Analyses from HARMONY, although limited by a small sample size, suggest no negative effect of pimavanserin on motor or cognition-related-function in patients with PD psychosis over up to 9 months of treatment. Rates of motor- and cognition-related TEAEs were similar between pimavanserin-treated and placebo-treated patients across studies. Pimavanserin did not exacerbate Parkinsonism and/or motor-related AEs, including orthostatic hypotension and falls; however, gait disturbance occurred at a higher rate in the pimavanserin group vs. placebo. Confusional state and cognitive disorder were the only 2 cognition-related TEAEs that occurred in patients in either the pooled analysis or in HARMONY in those treated with pimavanserin.

Results of a previous phase 2 study of pimavanserin in elderly patients with Alzheimer's disease psychosis have also shown no negative impact of pimavanserin on motor function and cognition (27). In a 10-year open-label extension study of pimavanserin in patients with PD psychosis, no occurrence of motor-related AEs beyond the expected progression of underlying PD with pimavanserin were reported (28).

The drug label for pimavanserin includes a black boxed warning indicating increased risk of mortality in elderly patients with dementia-related psychosis (26). In 2018, FDA had conducted a post-marketing analysis of reported mortality events and revealed no new or unexpected safety risks associated with pimavanserin; based on the post-marketing data, the FDA re-confirmed that the benefits of the drug outweigh the risks (29). Recent studies have reported increased long-term mortality in patients with PD taking pimavanserin (30) when compared to untreated PD patients.

There is an increased baseline mortality risk in patients with PDP, and conventional and atypical antipsychotics are also associated with an increased risk of mortality when used in elderly patients with PD with either psychosis or dementia (29, 30). Data from an open-label extension study of pimavanserin in PDP patients with a median duration of treatment of 454 days indicated a favorable safety profile over long-term treatment; there was also no increased risk of mortality (28). In addition, a recently published retrospective study of Medicare patients with PD who initiated treatment with pimavanserin or atypical antipsychotics suggest that, during the first 180 days of treatment, mortality rates in those taking pimavanserin were approximately 35% lower compared to patients taking atypical antipsychotics (31), while the risk of mortality was similar thereafter. Despite these findings, further studies to evaluate long-term mortality rates are warranted in this frail, elderly population.

Off-label use of antipsychotics and other medication in older adults is significantly associated with impaired activities of daily living and health-related quality of life (18). Motor-related AEs are of particular concern in patients with PD, for whom motor control is already compromised, and comorbid disease- and age-related impairments are common (32). Many antipsychotic medications used off label are antagonists at the dopamine D_2_ receptor and, therefore, can negatively impact motor function (17, 33, 34). Motor-related AEs associated with antipsychotics can mirror symptoms of PD, including akinesia, bradykinesia, and tremor (35), complicating management of PD symptoms. Altering dopaminergic medication regimens in patients with PD can be a balancing act in which psychotic symptoms may improve at the cost of worsening motor symptoms (14).

Quetiapine is the most commonly used antipsychotic to treat PD psychosis. While clinical trials have demonstrated a lack of motor worsening in patients taking quetiapine, there is no proven efficacy for addressing PDP. It is therefore likely that quetiapine is used to treat PDP due to improvements in secondary symptoms, such as sleep disturbance, agitation, and anxiety (36). In addition, studies have either shown no difference in mortality rates between pimavanserin and quetiapine (36) or lower rates of mortality in those taking pimavanserin compared to quetiapine (31, 37) for the first 180 days of use.

In patients with PD, development of hallucinations or delusions is associated with progression to dementia and cognitive decline (10, 38). The presence of hallucinations and/or delusions predicted both incidence of dementia and decline in MMSE score in patients with PD (10, 38). Atypical antipsychotic exposure has been shown to exacerbate this difference (13, 19). Here, there was no decline in MMSE score in patients with PD psychosis treated with pimavanserin 34 mg. Approximately 4–6% of patients who received pimavanserin in the pooled analysis or in the open-label period of HARMONY experienced a cognition-related TEAE. Although the sample size by week 26 of the double-blind period of HARMONY was small (pimavanserin, *n* = 4; placebo, *n* = 2), there was no decrease in MMSE score in patients treated with pimavanserin for the entire 9-month duration of the study.

MMSE was not assessed over the course of pimavanserin treatment in studies 012, 014, and 020, prohibiting analyses of cognitive effects of pimavanserin. In an analysis of study 020 outcomes by baseline MMSE score, pimavanserin improved psychosis symptoms in patients with PD regardless of baseline MMSE score or use of cognitive-enhancing medications (39). Pimavanserin improved the change from baseline to week 6 Scale for the Assessment of Positive Symptoms for Parkinson's Disease Psychosis score and Clinical Global Impressions—Global Improvement score, relative to placebo, in patients with cognitive impairment (baseline MMSE score, 21–24) and those without (baseline MMSE score, ≥25) and in patients with and without concomitant use of acetylcholinesterase inhibitors or memantine (39). Incidence of AEs was also similar across all 4 of these subgroups.

This analysis has several limitations. As the sample size calculations in HARMONY were based on efficacy measures rather than safety, Type II errors are possible. Therefore, statistical results were not included to avoid overstating the conclusions of the pooled analysis, in the context of Type II errors. The small sample sizes were based on the availability of data for PDP patients only from the clinical trials. Although data from HARMONY reflect longer-term pimavanserin exposure (i.e., up to 26 weeks), the short duration of pimavanserin exposure in studies in the pooled analysis would not reveal events that may occur with longer exposure. Observational studies, which can provide additional information on long-term exposure, were not taken into consideration here, as the lack of a comparator arm can make it difficult to assess motor and cognitive safety events relative to the placebo group (28).

Analysis of the subgroup of patients with PD dementia with psychosis treated with pimavanserin 34 mg in HARMONY limited the sample size, particularly at later time points in the double-blind period. Interpretation of the effect of pimavanserin on MMSE score must consider the possibility of a learning bias. In HARMONY, these tests were conducted at every visit (every 2–4 weeks), and the test was not presented in multiple forms to mitigate learning expected to arise from repeated testing. In addition, motor-related function and cognition were assessed as safety measures in all studies described here; these studies were not powered to detect differences between groups. While it is possible that a lack of motor worsening could present past the prespecified time points in these trials, long-term data of PDP patients taking pimavanserin indicated a safety profile consistent with results from the shorter-term clinical trials (28).

Treatment for PD psychosis should manage hallucinations and delusions without exacerbating underlying Parkinsonism, impairing cognition, or increasing the risk for sedation or other AEs that currently limit the use of antipsychotics. Overall, based on the collective data from 4 trials, pimavanserin was well tolerated and did not yield a negative impact on motor- or cognition-related function in elderly patients with PD psychosis.

## Data availability statement

The raw data supporting the conclusions of this article will be made available by the authors, without undue reservation.

## Ethics statement

Ethical review and approval was not required for the study on human participants in accordance with the local legislation and institutional requirements. Written informed consent from the patients/participants or patients/participants' legal guardian/next of kin was not required to participate in this study in accordance with the national legislation and the institutional requirements.

## Author contributions

VA, CBr, CBa, and AB contributed to the conception and design of the study. BC performed the statistical analysis. All authors contributed to the interpretation of the data for the manuscript and to manuscript revision and read and approved the submitted version of the manuscript.

## Funding

Acadia Pharmaceuticals Inc provided funding for this study.

## Conflict of interest

This study received funding from Acadia Pharmaceuticals Inc. The funder was involved in the study design, collection, analysis, interpretation of data, the writing of this article or the decision to submit it for publication. VA, AB, and BC are employees of Acadia Pharmaceuticals Inc. CBr was an employee of Acadia Pharmaceuticals Inc. at the time of the analyses. AE has received grant support from the NIH and the Michael J. Fox Foundation; personal compensation as a consultant/scientific advisory board member for AbbVie, Neuroderm, Neurocrine, Amneal, Acadia, Acorda, Avion Pharmaceuticals, Bexion, Herantis Pharma, Kyowa Kirin, Sunovion, Lundbeck, and Supernus (formerly, USWorldMeds); and publishing royalties from Lippincott Williams & Wilkins, Cambridge University Press, and Springer. He cofounded REGAIN Therapeutics and is owner of a patent application that covers synthetic soluble non-aggregating peptide analogs as a replacement treatment in proteinopathies. He serves on the editorial boards of the Journal of Parkinson's Disease, Journal of Alzheimer's Disease, European Journal of Neurology, Movement Disorders Clinical Practice, and JAMA Neurology. CBa has received grants and personal fees from Acadia and Lundbeck, and personal fees from Heptares, Roche, Lilly, Otsuka, Orion, GlaxoSmithKline, and Pfizer.

## Publisher's note

All claims expressed in this article are solely those of the authors and do not necessarily represent those of their affiliated organizations, or those of the publisher, the editors and the reviewers. Any product that may be evaluated in this article, or claim that may be made by its manufacturer, is not guaranteed or endorsed by the publisher.
