# Supplementary material for: Motor- and cognition-related safety of pimavanserin in patients with Parkinson's disease psychosis
Source: Front Neurol. 2022 Oct 5;13:919778. doi: 10.3389/fneur.2022.919778 (PMC9580496; doi:10.3389/fneur.2022.919778)
Supplement: Supplementary file 1 [file Table_1.DOCX]

**Supplemental Table 1.** Motor-related treatment-emergent adverse event preferred terms related to cognition

| **Group term: Extrapyramidal syndrome**  **Akathisia**  Akathisia  Extrapyramidal disorder  Motor dysfunction  Movement disorder  Psychomotor hyperactivity  Restlessness  **Dyskinesia**  Athetosis  Ballismus  Buccoglossal syndrome  Chorea  Choreoathetosis  Dopamine dysregulation syndrome  Dyskinesia  Dyskinesia esophageal  Grimacing  Oculogyric crisis  Pharyngeal dyskinesia  Protrusion tongue  Rabbit syndrome  Tardive dyskinesia  **Dystonia**  Dopa-responsive dystonia  Dystonia  Dystonic tremor  Emprosthotonus  Meige syndrome  Oculogyric crisis  Opisthotonus  Oromandibular dystonia  Pharyngeal dystonia  Spasmodic dysphonia  Torticollis  Trismus  **Parkinson-like events**  Akinesia  Bradykinesia  Cogwheel rigidity  Freezing phenomenon  Hypertonia  Hypertonia neonatal  Hypokinetic dysarthria  Muscle rigidity  On and off phenomenon  Parkinsonian crisis  Parkinsonian gait  Parkinsonian rest tremor  Parkinsonism  Parkinsonism hyperpyrexia syndrome  Propulsive gait  Resting tremor  Action tremor  Bradyphrenia  Drooling  Dysphonia  Extrapyramidal disorder  Fine motor skill dysfunction  Gait disturbance  Hypokinesia  Hypokinesia neonatal  Laryngeal tremor  Micrographia  Mobility decreased  Motor dysfunction  Movement disorder  Muscle tone disorder  Musculoskeletal stiffness  Postural reflex impairment  Postural tremor  Reduced facial expression  Tremor  Walking disability |
| --- |
| **Group term: Sedation-like events**  Sedation  Somnolence  Fatigue  Asthenia  Lethargy  Hypersomnia |
