# Supplementary material for: Motor- and cognition-related safety of pimavanserin in patients with Parkinson's disease psychosis
Source: Front Neurol. 2022 Oct 5;13:919778. doi: 10.3389/fneur.2022.919778 (PMC9580496; doi:10.3389/fneur.2022.919778)
Supplement: Supplementary file 2 [file Table_2.DOCX]

**Supplemental Table 2.** Cognition-related treatment-emergent adverse event preferred terms related to cognition

| **Group term: Cognitive and attention disturbances**  Borderline mental impairment  Cerebellar cognitive affective syndrome  Change in sustained attention  Cognitive disorder  Cognitive linguistic deficit  Daydreaming  Distractibility  Disturbance in attention  Executive dysfunction  Mental fatigue  Mental impairment  Psychomotor disadaptation syndrome  Vascular cognitive impairment |
| --- |
| **Group term: Psychiatric disorders**  Confusional state |
